# Role of the SaeRS two-component regulatory system in *Staphylococcus epidermidis *autolysis and biofilm formation

**DOI:** 10.1186/1471-2180-11-146

**Published:** 2011-06-24

**Authors:** Qiang Lou, Tao Zhu, Jian Hu, Haijing Ben, Jinsong Yang, Fangyou Yu, Jingran Liu, Yang Wu, Adrien Fischer, Patrice Francois, Jacques Schrenzel, Di Qu

**Affiliations:** 1 Key laboratory of Medical Molecular Virology of Ministry of Education and Ministry of Public Health, Institute of Medical Microbiology and Institutes of Biomedical Sciences, Shanghai Medical College of Fudan University, 138 Yixueyuan Road, Shanghai, 200032, PR China; 2 Laboratory of Cellular and Molecular Immunology, Henan University, Jinming Road, Kaifeng, 475004, PR China; 3 Department of Orthopedics, The First Affiliated Hospital of Guangxi Medical University, 6 Shuangyong Road, Nanning, 530021, PR China; 4 Department of Laboratory Medicine, the First Affiliated Hospital of Wenzhou Medical College, 2 Fuxue Road, Wenzhou, 325000, PR China; 5 Genomic Research Laboratory, Service of Infectious Diseases, University of Geneva Hospitals, Rue Gabrielle-Perret-Gentil 4, Geneva, CH-1211, Switzerland

## Abstract

**Background:**

*Staphylococcus epidermidis *(SE) has emerged as one of the most important causes of nosocomial infections. The SaeRS two-component signal transduction system (TCS) influences virulence and biofilm formation in *Staphylococcus aureus*. The deletion of *saeR *in *S. epidermidis *results in impaired anaerobic growth and decreased nitrate utilization. However, the regulatory function of SaeRS on biofilm formation and autolysis in *S. epidermidis *remains unclear.

**Results:**

The *saeRS *genes of SE1457 were deleted by homologous recombination. The *saeRS *deletion mutant, SE1457*ΔsaeRS*, exhibited increased biofilm formation that was disturbed more severely (a 4-fold reduction) by DNase I treatment compared to SE1457 and the complementation strain SE1457*saec*. Compared to SE1457 and SE1457*saec*, SE1457*ΔsaeRS *showed increased Triton X-100-induced autolysis (approximately 3-fold) and decreased cell viability in planktonic/biofilm states; further, SE1457*ΔsaeRS *also released more extracellular DNA (eDNA) in the biofilms. Correlated with the increased autolysis phenotype, the transcription of autolysis-related genes, such as *atlE *and *aae*, was increased in SE1457*ΔsaeRS*. Whereas the expression of accumulation-associated protein was up-regulated by 1.8-fold in 1457*ΔsaeRS*, the expression of an N-acetylglucosaminyl transferase enzyme (encoded by *icaA*) critical for polysaccharide intercellular adhesin (PIA) synthesis was not affected by the deletion of *saeRS.*

**Conclusions:**

Deletion of *saeRS *in *S. epidermidis *resulted in an increase in biofilm-forming ability, which was associated with increased eDNA release and up-regulated Aap expression. The increased eDNA release from SE1457*ΔsaeRS *was associated with increased bacterial autolysis and decreased bacterial cell viability in the planktonic/biofilm states.

## Background

The opportunistic pathogen *Staphylococcus epidermidis *has emerged as an important etiologic agent of nosocomial infections. The ability to form biofilms on the surfaces of medical devices is an important component of *S. epidermidis *pathogenicity. Biofilm resistance to antibiotics and host defense mechanisms are often regulated by two-component signal transduction systems (TCSs) [1].

Biofilm formation proceeds in two distinct developmental phases: primary attachment of staphylococcal cells to a polystyrene surface followed by bacterial accumulation in multiple layers [2]. The initial adhesion of bacterial cells to a polymer surface is influenced by a variety of factors, including AtlE, Embp, and other staphylococcal surface-associated proteins. During the bacterial accumulation phase in *S. epidermidis*, biofilm formation is mediated by extracellular polysaccharides and proteins, such as polysaccharide intercellular adhesin (PIA) [3] and accumulation-associated protein (Aap) [4]. In addition to extracellular polysaccharides and proteins, extracellular DNA (eDNA) is a matrix component that is critical for bacterial attachment during the initial stage of biofilm formation [5,6]. Extracellular DNA release from *S. epidermidis *is related to AtlE-mediated bacterial autolysis [7]. Another autolysin recently identified in *S. epidermidis*, Aae, also has bacteriolytic activities and adhesive properties [8].

TCSs regulate bacterial adaptation, survival, virulence and biofilm formation [9-12]. TCSs comprise a membrane-associated histidine kinase and a cytoplasmic response regulator. Overall, 16 or 17 TCSs have been identified in the genomes of *S. epidermidis *ATCC12228 or ATCC35984 [13,14]. In *S. epidermidis*, the TCS *agrC/agrA *has been proven to negatively regulate biofilm formation [15,16]. In a previous study of the *S. epidermidis saeRS *TCS, a *saeR *deletion mutant exhibited a lower anaerobic growth rate, a significantly reduced rate of nitrate utilization and a slightly higher biofilm-forming ability compared to the parental strain [11]. In *S. aureus*, the *saeRS *TCS influences biofilm formation [17] and the expression of virulence-associated factors, such as protein A, α- and β-hemolysins, and coagulase [18]. However, whether *saeRS *regulates *S. epidermidis *autolysis and biofilm formation remains unclear.

In the present work, we constructed a SE1457*ΔsaeRS *mutant with deletion of the genes that encode both the histidine kinase (SaeS) and the response regulator (SaeR) by homologous recombination. The effects of the *saeRS *deletion on *S. epidermidis *autolysis, eDNA release, bacterial cell viability, and biofilm formation were investigated.

## Methods

### Bacterial strains, plasmids, and media

The bacterial strains and plasmids used in this study are listed in Table 1. *S. epidermidis *cells were grown at 37°C in BM medium (per liter = tryptone 10 g, yeast extract 5 g, NaCl 5 g, K2HPO4 1 g, and glucose 1 g) or tryptic soy broth (TSB) (Oxiod, Basingstoke, Hampshire, England) supplemented with antibiotics when necessary. Antibiotics were used at the following concentrations: erythromycin at 2.5 μg/mL, chloramphenicol at 10 μg/mL, spectinomycin (spc) at 300 μg/mL for *S. epidermidis *and *S. aureus*, and ampicillin at 100 μg/mL for *E.coli*.

**Table 1:** Bacterial strains and plasmids used in the present study

Strain or plasmid	Relevant genotype or characteristic	Reference or source
**Strains**		
*E. coli *DH5α	λ^- ^ϕ80d*lac*Δ*M15 *Δ(*lacZYA-argF*)*U169 recA1 endA1 hsdR17 *(rK^- ^mK^-^) *supE44 thi-1 gyrA relA1*	[49]
SE1457	Biofilm positive strain	[50]
*S. aureus RN4220*	Restriction-negative, modification-positive isolate	[51]
SE1457*ΔsaeRS*	*saeRS *deletion mutant of strain 1457, Spc^r^	This study
SE1457*saec*	1457*ΔsaeRS *complemented with *saeRS*	This study
**Plasmids**		
pET-28a(+)	Expression vector, Kan^R^	Novagen
pBT2 pCX19	Temperature-sensitive *E. coli- Staphylococcus *shuttle vector. Ap^r ^(*E. coli*) Cm^r ^(*Staphylococcus*) Derivate of pCX15	[52] [53]
pMAD	*Escherichia coli/Staphylococcus *Shuttle vector	[54]
pMAD-*saeRS*	Vector for allelic gene replacement of *saeRS *in *S. epidermidis*	This study
pBT2-*saeRS*	Vector for complementation of *saeRS *in *S. epidermidis *1457*ΔsaeRS*	This study

*Abbreviations: Amp, ampicillin; Cm, chloramphenicol; Em, erythromycin.

### Determination of the growth curves of *S. epidermidis *strains

The aerobic growth curves of *S. epidermidis *strains were determined by measuring the optical density (OD600) as described previously [11]. Briefly, overnight cultures were diluted 1:200 and incubated at 37°C with shaking at 220 rpm. The OD600 of the culture were measured at 60 min intervals for 12 h. At 6, 12, and 24 h time points, colony forming units on TSA plates were further counted with serial dilutions of each sample plated on 6 agar plates. For anaerobic growth conditions, bacteria were cultured in the Eppendorf tubes which were filled up with the TSB medium and sealed with wax.

### Detection of biofilm formation

The biofilm-forming ability of *S. epidermidis *strains was determined by the microtiter-plate test as described by Christensen [19,20]. Briefly, overnight cultures of *S. epidermidis *were diluted 1:200 and inoculated into wells of polystyrene microtiter plates (200 μL per well) at 37°C for 24 h. At different time points (0, 6, 12, and 24 h), DNase I (Takara Bio, Kyoto, Japan) was added at 28 U/200 μL. After incubation, the wells were gently washed three times with 200 μL PBS and stained with 2% crystal violet for 5 min. Absorbance was determined at 570 nm.

To determine whether *saeRS *affects cell death in biofilms, *S. epidermidis *cells were cultivated in FluoroDish (FD35-100, WPI, USA) as previously described [7]. Briefly, overnight cultures of *S. epidermidis *grown in TSB medium were diluted 1:200, inoculated into dishes (2 mL per dish), and then incubated at 37°C for 24 h. The dishes were then carefully washed with PBS and stained with a LIVE/DEAD kit (containing SYTO9 and PI, Invitrogen Molecular Probes, USA) following the manufacturer's instructions. SYTO9 stains viable bacteria green while PI stains dead bacteria red. Biofilms of *S. epidermidis *1457 and SE1457*ΔsaeRS *were observed under a Leica TCS SP5 confocal laser scanning microscope (CLSM) using a 63 ×(zoom ×3) objective lens and the Z-stack composite confocal photomicrographs of viable cells, dead cells, and both cells (viable & dead) were generated by Leica LAS AF softwear (version 1.8.1). The fluorescence quantity of each stack was determained using ImageJ software.

### Electron microscopy

For scanning electron microscopy (SEM), biofilms were grown in TSB for 24 h at 37°C with fragments of an introvenous catheter, rinsed with PBS three times, fixed with a 2% (w/v) solution of glutaraldehyde prepared in phosphate-buffered saline, and then observed under a TECNAI- 12 field emission source instrument (Philips, Eindhoven, The Netherlands).

For transmission electron microscopy (TEM), bacteria grown for 24 h were stained by mixing with a 1% (w/v) solution of uranyl acetate on an electron microscope grid covered with a carbon-coated Formvar film. *S. epidermidis *cells were observed using a Hitachi S-520 electron microscope (Hitachi, Tokyo, Japan).

### RNA extraction and microarray analysis

Overnight cultures of *S. epidermidis *1457 and 1457 *ΔsaeSR *were diluted 1:200 into fresh TSB and grown at 37°C to an OD600 of 3.0 (mid-exponential growth). Eight millilitres of bacterial cultures were pelleted, washed with ice-cold saline, and then homogenized using 0.1 mm Ziconia-silica beads in Mini-Beadbeater (Biospec) at a speed of 4800 rpm. The bacterial RNA was isolated using a QIAGEN RNeasy kit according to the standard QIAGEN RNeasy protocol.

The microarray was manufactured by in situ synthesis of 14,527, 60-mer long oligonucleotide probes (Agilent, Palo Alto, CA, USA), selected as previously described [21]. It covers > 95% of all ORFs annotated in strains ATCC12228 (GeneBank accession number NC_004461), ATCC35984 (GeneBank accession number NC_002976), SE1457 (unpublished sequence). Preparations of 10 μg of total *S. epidermidis *RNA were labeled by Cy-3 dCTP (Perkin-Elmer) using the SuperScript II (Invitrogen, Basel, Switzerland) and purified as previously described [22]. Pool of purified genomic DNA from the reference sequenced strains used for the design of the microarray was labeled with Cy-5 dCTP [21] and used for microarray normalization [23]. Mixtures of Cy5-labeled DNA and Cy3-labeled cDNA were hybridized and scanned as previously described [22] in a dedicated oven. Fluorescence intensities were quantified using Feature Extraction software (Agilent, version 8). Green (Cy3) and red (Cy5) feature extraction processed data were imported in the Partek genomics suite software (Partek Incorporated. St. Louis, USA). Data were normalized to baseline using red channel data as control [23] and mean to estimate baseline. Variance analysis of three biological replicates was processed with a false discovery rate value of 5% (P value cutoff; 0.05) and an arbitrary threshold of 3.0 fold for defining significant differences in expression ratios. The complete raw microarray dataset has been posted on the Gene Expression Omnibus database (http://www.ncbi.nlm.nih.gov/geo/), accession number GPL13532 for the platform design and GSE29309 for the original dataset.

### Quantitative real-time PCR analysis

DNase-treated RNA was reverse transcribed using M-MLV and a hexamer random primer mix. Appropriate concentration of cDNA sample was then used for real-time PCR using an ABI 7500 real-time PCR detection system, gene-specific primers, and the SYBR Green I mixture (Takara, Dalian, China). Relative expression levels were determined by comparison to the level of *gyrB *expression in the same cDNA preparations. Gene-specific primers were designed according to GenBank gene sequences (Accession number: CP000029, Table 2). All samples were analyzed in triplicate and normalized against *gyrB *expression.

**Table 2:** Oligonucleotide primers

Target gene	GenBank accession no.	Primer*	Primer sequence	Location
**Oligonucleotide primers used for RT real-time PCR**				
*gyrB*	57636585	gyrB-F	CTTATATGAGAATCCATCTGTAGG	1110-1263
		gyrB-R	AGAACAATCTGCCAATTTACC	
*lrgA*	57636056	lrgA-F	TGGACTTGTACTATTATTTATCGC	165-309
		lrgA-R	AAGGATTGGTAAAGAGTTAATGAC	
*lytS*	57636054	lytS-F	CTGTTCAAGATAATGGTCAAGG	1535-1680
		lytS-R	CAGTGCCGATGTTGTTCC	
*serp0043*	57636640	serp0043-F	CAAGCACAAGCGTCTTCATC	73-236
		serp0043-R	ACTCTTTCACCATTATTTGTTTCAG	
*glpQ*	57637130	glpQ-F	CCGTTACACTGGGTTTAGC	41-221
		glpQ-R	TTACCACTTACTGAGTCTGATTC	
*arlR*	57636010	arlR-F	AGAGAATGATGGAAAGGCAGGT	90-253
		arlR-R	ATGTCTCGCTTTTCGCAGTAAT	
*atlE*	57637180	atlE-F	AACAACCACAGAATCAGTCTAATC	92-237
		atlE-R	TTGAACTTGGGTAGGGTCTTG	
*aae*	57637180	aae-F	AACAAATTGATAAAGCAACG	1970-2186
		aae-R	GTTGTCTTTCCTTTAGTGTC	
*aap*	57636451	aap-F	AATAGAACCTACAACTTCAGAACC	945-1039
		aap-R	TGTTATTGGATGAACTATCAGCAG	
*icaA*	57636387	icaA-F	GGTTGTATCAAGCGAAGTC	556-754
		icaA-R	ACATCCAGCATAGAGCAC	
*saeS*	57636974	saeS-F	GGTATCGTTCCAGAACTTCAATC	757-881
		saeS-R	ATTTGTTGTGCTAACTCATTTGC	
*saeR*	57636975	saeR-F	CTCAAGAACATGACACGATATACG	245-354
		saeR-R	TCTAGCGAGAAGGTTATTAGTACG	
*saeQ*	57636990	saeQ-F	GCAAGTTTCTTTGGAGCCTTC	268-447
		saeQ-R	CTTATCTTCACCTCGGTTATTACG	
*saeP*	57636991	saeP-F	CTAACTCGGAAAGCGATCAC	71-258
		saeP-R	GTCTGGACCTTTAGAAGATTTG	
**Oligonucleotide primers used for eDNA quantification**				
*gyrA*	57636584	gyrA-F	CCTTATGAAACTCGGAGATGG	2382-2489
		gyrA-R	TCAGTAGTAGTAGATTGTTGCG	
*lysA*	57637514	lysA-F	TGACAATGGGAGGTACAAGC	32-107
		lysA-R	TGGTCTTCATCGTAAACAATCG	
*serp0306*	57636873	serp0306-F	ATGCCACATCCACGAAAGA	203-381
		serp0306-R	TGTAACTGACAATGCCCAATC	
*leuA*	57638228	leuA-F	GTGAACGGTATTGGTGAAAGAG	685-762
		leuA-R	GTGGTCCTTCCTTACATATAAAGC	

F, forward primer; R, reverse primer

### Determination of Triton X-100-induced autolysis

Triton X-100-induced autolysis was performed to determine the potential role of *saeRS *in autolysis regulation in *S. epidermidis*, as described elsewhere [24-26]. SE1457*ΔsaeRS*, SE1457, and SE1457*saec *cells were diluted in TSB containing 1 M NaCl, grown to mid-exponential phase (OD600 = ~0.6-0.8), washed twice in cold sterile distilled water, resuspended in the same volume of 0.05 M Tris-HCl containing 0.05% Triton X-100 (pH 7.2), and incubated at 30°C. OD600 was measured every 30 min. The Triton X-100-induced autolysis rate was calculated as follows: Ra = OD0-ODt/OD0.

### Zymogram

The murein hydrolase activities of SE1457, SE1457*ΔsaeRS*, SE1457*saec*, and SE1457*ΔatlE *were detected by zymographic analysis as described elsewhere [26,27]. Extracts from lysostaphin- and SDS-treated *S. epidermidis *(Ex-Lys and Ex-SDS, respectively) and the concentrated supernatants of the bacterial culture (Ex-Sup) were used to analyze the murein hydrolase activities of each strain. Ex-Lys were obtained by treating *S. epidermidis *cells with 30 μg/mL of lysostaphin for 2 h at 37°C and subsequently centrifuged at 8,000 *g *for 30 min. Ex-SDS were obtained by treating *S. epidermidis *cells in 100 μL of 100 mM phosphate buffer containing 4% SDS at 37°C for 30 min and centrifuged (10,000 *g*) for 10 min. Ex-Sup were acquired by concentrating supernatants of overnight *S. epidermidis *cultures to 10% initial volume using a centrifugal filter device (Millipore, Billerica, MA).

*S. epidermidis *cell extracts were separated on a SDS-PAGE gel (10% acrylamide, pH 8.8) containing 0.2% (wt/vol) lyophilized *Micrococcus luteus *(*M. luteus*) or *S. epidermidis *cells. After electrophoresis, the gels were washed four times with distilled water for 30 min at room temperature, incubated in 25 mM Tris-HCl containing 1% Triton X-100 (pH 8.0) at 37°C for 6 h, and then stained with methylene blue.

### Quantification of eDNA

Extracellular DNA isolation from biofilms was performed as described by Rice *et al*. [7,19,28]. Briefly, SE1457, SE1457*ΔsaeRS*, and SE1457*saec *biofilms (grown for 24 h) were chilled at 4°C for 1 h and treated with 1.0 μL of 0.5 M EDTA. Supernatants were discarded, and the unwashed biofilms were resuspended in 50 mM TES buffer (Tris-HCl (pH 8.0), 10 mM ETDA, 500 mM NaCl). Extracellular DNA was extracted with phenol/chloroform/isoamyl alcohol (25:24:1), precipitated with 100% ethanol, and dissolved in 20 μL of TE buffer.

Extracellular DNA was quantified by qPCR using *gyrA *(gyrase A), *serp0306 *(ferrichrome transport ATP-binding protein A), *lysA *(diaminopimelate decarboxylase A), and *leuA *(2-isopropylmalate synthase) primers as listed in Table 2. Each sample was diluted to 1:10, and PCRs were performed with SYBR *Premix Ex Taq^TM ^*(TaKaRa, Japan) and primers (2 μM), according to the manufacturer's recommendations. The average OD600 of each unwashed biofilm was determined for calculating potential differences in biomass. The amount of eDNA per relative biomass of each biofilm was then calculated as follows: total eDNA (ng)/ relative OD600.

### Initial bacterial attachment assays

Initial cell attachment was detected as described by Heilmann *et al*. [29]. Briefly, mid-exponential phase cells were diluted to OD600 = 0.1 in PBS and then incubated in wells (1 mL per well) of cell-culture polystyrene chambers (Nunc, Denmark) with DNase I (140 U/mL) for 2 h at 37°C. Numbers of attached cells were counted under a microscope. Three independent experiments were carried out.

### Detection of Aap expression

Concentrations of lysostaphin-treated whole bacterial proteins from SE1457*ΔsaeRS*, SE1457, and SE1457*saec *were determined by the Bradford method. For the detection of Aap in all samples by Western blot assay, proteins were separated on a 7% SDS-PAGE gel and then transferred to polyvinylidene fluoride (PVDF) membranes (Whatman, D-37586 Dassel, Germany) by electroblotting with a Mini-Transfer system (Bio-Rad, Mississauga, Canada) at 200 mA for 2 h (4°C). Monoclonal antibodies against the Aap B-repeat region (prepared by Abmart, Shanghai, China) were diluted 1:6000, and horseradish peroxidase-conjugated goat anti-mouse IgG antibodies (Sino-American Biotech) were diluted 1:2000. The gray scale of the bands corresponding to Aap was quantified using the Quantity-one software (Bio-Rad, USA).

### Semi-quantitative detection of PIA

PIA was detected as described elsewhere [30-32]. Briefly, *S. epidermidis *strains were grown in 6-well plates (Nunc, DK-4000 Roskitde, Denmark) under static conditions at 37°C for 24 h. The cells were scraped off and resuspended in 0.5 M EDTA (pH 8.0). The supernatant was treated with proteinase K (final concentration 4 mg/mL; Roche, MERCK, Darmstadt, Germany) for 3 h (37°C). Serial dilutions of the PIA extract were then transferred to a nitrocellulose membrane (Millipore, Billerica, MA) using a 96-well dot blot vacuum manifold (Gibco). The air-dried membrane was blocked with 3% (wt/vol) bovine serum albumin and subsequently incubated with 3.2 μg/mL wheat germ agglutinin coupled to horseradish peroxidase (WGA-HRP conjugate; Lectinotest Laboratory, Lviv, Ukraine) for 1 h. Horseradish peroxidase (HRP) activity was visualized via chromogenic detection. The gray scale of the spots corresponding to PIA was quantified using the Quantity-one software.

### Statistical analysis

Experimental data were analyzed with the SPSS software and compared using the Student's *t*-test. Differences with a P value of < 0.05 were considered statistically significant.

## Results

### Effect of *saeRS *deletion on *S. epidermidis *biofilm formation

**Figure 1:**
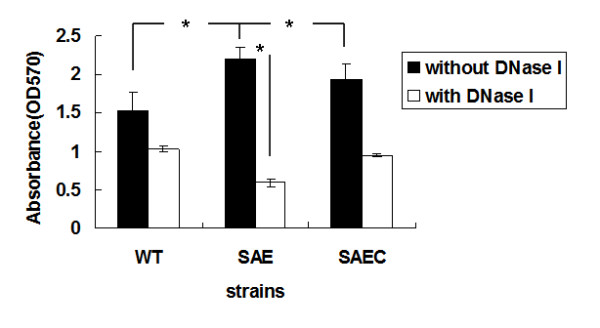
**Effect of DNaseI on SE1457*ΔsaeRS*, SE1457, and SE1457*saec *biofilm formation**. SE1457*ΔsaeRS*, SE1457, and SE1457*saec *biofilms were washed and then stained with crystal violet. Their retained biomass was quantified by measuring the absorbance of each well at 570 nm. Biofilms were formed in the absence (black bars) or presence of DNase I (28 U/200 μL/well) (white bars). Mean values and standard deviations from three independent experiments are shown. (*), P < 0.05. WT, SE1457; SAE, SE1457*ΔsaeRS*; SAEC, SE1457*saec*.

In order to explore the influence of *saeR *and *saeS *on *S. epidermidis *biofilm formation, an *S. epidermidis *1457*ΔsaeRS *mutant (SE1457*ΔsaeRS*) and a complemented strain (SE1457*saec*) were constructed using the shuttle plasmids pMAD and pBT2, respectively. The biofilm-forming ability of SE1457*ΔsaeRS *on polystyrene plates was higher compared to the parental strain. Although it did not reach the level of the wild-type strain, complementation of *saeRS *resulted in decreased biofilm formation (Student's *t*-test, P < 0.05) (Figure 1). The growth curves of SE1457*ΔsaeRS *and the parental strain were similar in either aerobic or anaerobic growth conditions (Additional file 1: Fig. S1).

**Figure 2:**
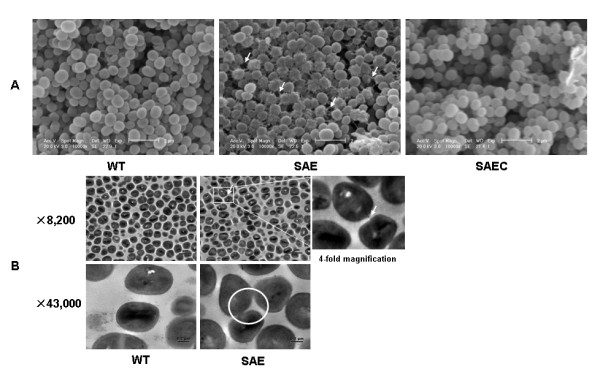
**SEM and TEM observations of SE1457*ΔsaeRS *and wild-type strain**. (A) Biofilms of SE1457*ΔsaeRS*, SE1457, and SE1457*saec *after 24 h of growth on hydroxyapatite disks were observed by SEM. Arrows show the extracellular polymeric substances (EPSs) (10,000× magnification). (B) Planktonic cells of SE1457*ΔsaeRS *and SE1457 cultured for 24 h were observed by TEM. Cell-cell accumulations in SE1457*ΔsaeRS *are circled; arrow indicates the thread-like material linking neighboring cells. WT, SE1457; SAE, SE1457*ΔsaeRS*; SAEC, SE1457*saec*.

Scanning electron microscopy (SEM) of biofilms on catheters showed that SE1457*ΔsaeRS *biofilms contained more extracellular matrix compared to SE1457 and SE1457*saec *biofilms (Figure 2A). In planktonic cultures, intercellular adhesion of the SE1457*ΔsaeRS *and the wild-type strain was observed using transmission electron microscopy (TEM). While thread-like material between SE1457*ΔsaeRS *cells was observed, such material was rarely found between parental strain cells (Figure 2B).

### Effect of *saeRS *deletion on the autolysis of *S. epidermidis*

**Figure 3:**
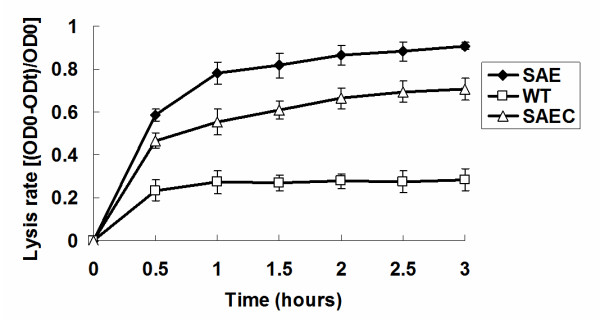
**Effect of *saeRS *deletion on Triton X-100-induced autolysis**. SE1457*ΔsaeRS*, SE1457, and SE1457*saec *cells were diluted in TSB medium containing 1 M NaCl, grown to mid-exponential phase (OD600 = ~0.6-0.8), and resuspended in the same volume of 0.05 M Tris-HCl solution containing 0.05% Triton X-100 (pH 7.2). OD600 readings were measured every 30 min. The autolysis rate induced by Triton X-100 was calculated as follows: lysis rate = OD0 - ODt/OD0. The experiments were carried out in triplicate independently. WT, SE1457; SAE, SE1457*ΔsaeRS*; SAEC, SE1457*saec.*

To examine the effect of *saeRS *deletion on autolysis, Triton X-100-induced autolysis of SE1457*ΔsaeRS*, SE1457, and SE1457*saec *was analyzed. Bacterial cells were harvested at the mid-exponential phase grown in TSB medium containing 1 M NaCl. Following the addition of 0.05% Triton X-100, SE1457*ΔsaeRS *cultures exhibited a much higher autolysis rate (approximately 3-fold) compared to the wild-type strain; decreased autoloysis was partially restored in the complementation strain SE1457*saec *(Figure 3).

**Figure 4:**
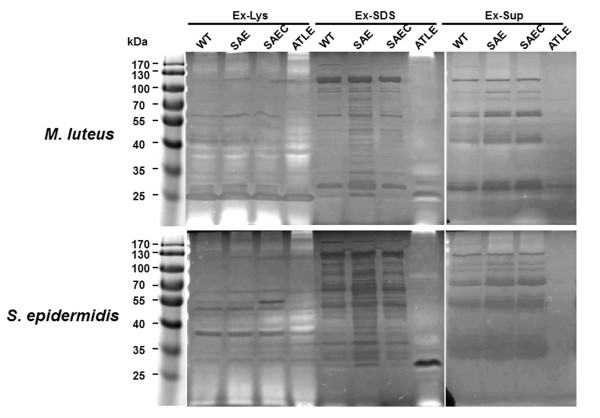
**Zymographic analysis of autolytic enzyme extracts**. Bacteriolytic enzyme profiles were analyzed on SDS gels (10% separation gel) containing lyophilized *M. luteus *cells (0.2%) or *S. epidermidis *cells (0.2%) as substrates. After electrophoresis, the gels were washed for 30 min in distilled water, incubated for 6 h at 37°C in a buffer containing Triton X-100, and then stained with methylene blue. The *S. epidermidis atlE *mutant was used as a negative control. Bands with lytic activity were observed as clear zones in the opaque gel. The clear zones appeared as dark bands after photography against a dark background. The molecular mass standard is shown on the left of the gels. Ex-Lys, cell-wall extracts of lysostaphin-treated *S. epidermidis*; Ex-SDS, cell-wall extracts of SDS-treated *S. epidermidis*; Ex-Sup, concentrated *S. epidermidis *culture supernatants; WT, SE1457; SAE, SE1457*ΔsaeRS*; SAEC, SE1457*saec*; ATLE, SE1457*ΔatlE*.

The effect of *saeRS *deletion on murein hydrolase activity was determined by zymographic analysis using lyophilized *Micrococcus luteus *(*M. luteus*) or *S. epidermidis *cells as substrates [26,33]. Briefly, extracts from lysostaphin- and SDS-treated *S. epidermidis *(Ex-Lys and Ex-SDS, respectively) cells and concentrated supernatants of the bacterial culture (Ex-Sup) were used to assess the murein hydrolase activities of each strain. As a control, extracts from the *S. epidermidis atlE *deletion mutant SE1457*ΔatlE *were used and resulted in only one lytic band (~30 kDa). In contrast, extracts from SE1457, SE1457*ΔsaeRS *and SE1457*saec *displayed multiple bacteriolytic bands. The zymogram profiles of Ex-SDS from SE1457*ΔsaeRS *extracts showed more lytic bands (from 25 to 90 kDa) compared to the zymogram profiles of SE1457 and SE1457*saec *extracts, indicating that autolysins may contribute to the increased autolysis of the mutant strain. The Ex-Lys and Ex-Sup zymogram profiles of SE1457*ΔsaeRS *were similar to the profiles observed for SE1457 and SE1457*saec *(Figure 4).

### Effect of *saeRS *deletion on *S. epidermidis *viability in planktonic and biofilm states

**Figure 5:**
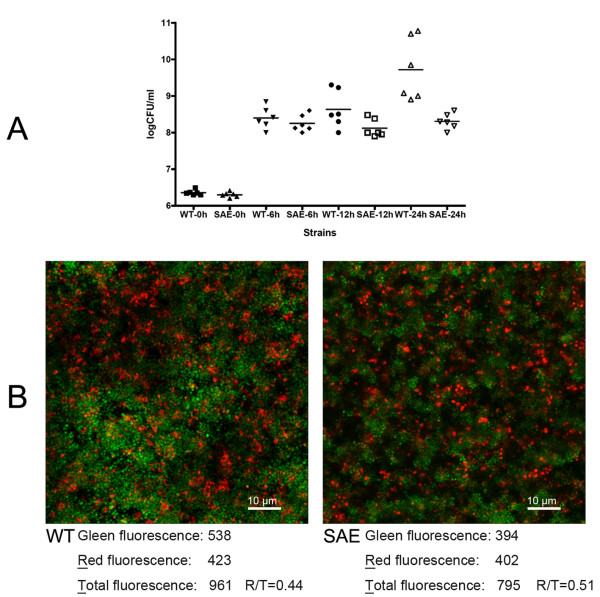
**Viability of *S. epidermidis *1457 in biofilms and the planktonic state**. (A) CFU counts of SE1457*ΔsaeRS *and SE1457. After 0, 6, 12, and 24 h of incubation, CFUs for SE1457 and SE1457*ΔsaeRS *cultures were calculated using serial dilutions of each sample plated on 6 agar plates. (B) CLSM images of *S. epidermidis *biofilms. SE1457 and SE1457*ΔsaeRS *were incubated in glass-bottomed cell culture dishes. After incubation at 37°C for 24 h, SE1457*ΔsaeRS *and SE1457 cells in biofilms were stained with LIVE/DEAD reagents that indicate viable cells by green fluorescence (SYTO9) and dead cells by red fluorescence (PI). Results depict a stack of images taken at approximately 0.3 μm depth increments and represent one of the three experiments. Fluorescence intensities were quantified using ImageJ software. WT, SE1457; SAE, SE1457*ΔsaeRS.*

To investigate whether the increased autolysis that resulted from *saeRS *deletion affected *S. epidermidis *cell viability, colony-forming unit (CFU) counts of the SE1457 and SE1457*ΔsaeRS *strains in the planktonic state were determined. Cultures were inoculated with approximately 10^4 ^CFU/mL of each strain and incubated under normal conditions. At 6 h, SE1457*ΔsaeRS *and SE1457 had log CFU/mL counts of 8.2 of and 8.4, respectively. CFU counts were also similar at 12 h post-inoculation, with log CFU/mL counts of 8.1 and 8.6 for SE1457*ΔsaeRS *and SE1457 respectively. However, after 24 h, SE1457*ΔsaeRS *cultures had a lower CFU count (8.3 log CFU/mL) compared to the wild-type strain (9.7 log CFU/mL) (P = 0.002) (Figure 5A).

The viability of SE1457*ΔsaeRS *and the wild-type strain in 24 h biofilm was determined by confocal laser scanning microscopy (CLSM) with LIVE/DEAD staining [34]. More dead cells were observed in the SE1457*ΔsaeRS *biofilm compared to the wild-type strain (Figure 5B).

### Effect of *saeRS *deletion on eDNA release from *S. epidermidis*

**Figure 6:**
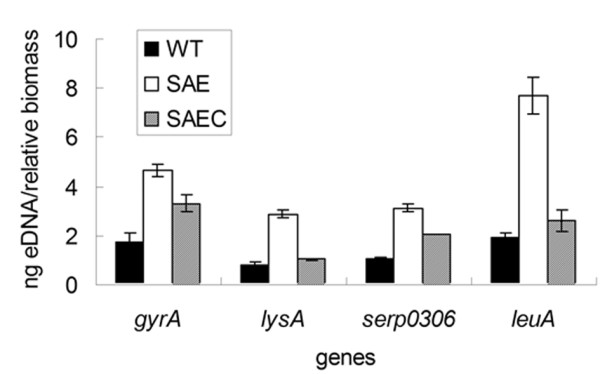
**Quantification of eDNA in SE1457*ΔsaeRS*, SE1457, and SE1457*saec *biofilms**. eDNA was extracted from the unwashed 24 h biofilms of SE1457*ΔsaeRS *(white bars), SE1457 (black bars), and SE1457*saec *(gray bars). The eDNA in each biofilm was quantified by qPCR using primers specific for *gyrA, serp0306, lysA*, and *leuA *[19,28]. The quantity of eDNA was calculated as follows: total eDNA (ng)/relative OD600. Results represent the mean ± SD of three independent experiments. WT, SE1457; SAE, SE1457*ΔsaeRS*; SAEC, SE1457*saec.*

Extracellular DNA is an important component of the *S. epidermidis *biofilm matrix [7,35], and its relative concentration in 24 h biofilms formed by SE1457, SE1457*ΔsaeRS *and SE1457*saec *was measured utilizing qPCR for *gyrA, lysA, serp0306*, and *leuA *[19,28]. Extracellular DNA concentrations were increased in the SE1457*ΔsaeRS *biofilms compared to the complementation strain and the wild-type strain (Figure 6).

When DNase I (28 U/200 μL/well) was added prior to biofilm formation, the biomass of the SE1457*ΔsaeRS *biofilms was decreased by 4-fold (P < 0.05); in contrast, the biomasses of SE1457 and SE1457*saec *biofilms were decreased by 1.5-fold (Figure 1).

### Effect of eDNA release on SE1457*ΔsaeRS *primary attachment of SE1457*ΔsaeRS*

**Figure 7:**
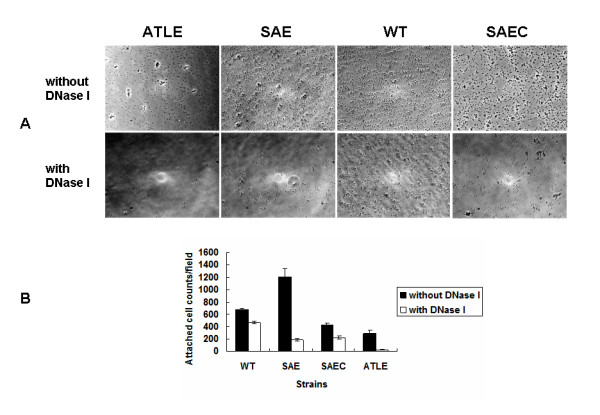
***S. epidermidis *attachment to polystyrene surfaces in the presence or absence of DNase I**. (A) Attached SE1457*ΔatlE*, SE1457*ΔsaeRS*, SE1457 and SE1457*saec *cells were observed by microscopy. Briefly, cell suspensions from the mid-exponential phase were diluted to OD600 = 0.1 in PBS and then incubated in wells (1 mL per well) of cell-culture polystyrene chambers (Nunc, Roskilde, Denmark) with DNase I (140 U/mL) for 2 h at 37°C. *S. epidermidis *cells attached to the polystyrene surface were counted under microscope (400× magnification). (B) The number of attached bacteria per field was then counted. Results represent the mean ± SD of three independent experiments. *, P < 0.05; WT, SE1457; SAE, SE1457*ΔsaeRS*; SAEC, SE1457 *saec*; ATLE, SE1457*ΔatlE.*

Extracellular DNA is a critical component for bacterial adhesion during the initial stage of biofilm development [5,6]. *S. epidermidis *cells attached to the polystyrene surface were counted under a microscope at 400× magnification. While 6.8 × 10^2^, 1.2 × 10^3^, and 4.2 × 10^2 ^cells per field were adhered for SE1457, SE1457*ΔsaeRS*, and SE1457*saec *strains, respectively, few attached SE1457*ΔatlE *cells were observed. When DNase I (140 U/mL) was added at the time of the attachment assay, SE1457*ΔsaeRS *cell attachment was significantly reduced by 85%. In contrast, following DNase I addition SE1457 and SE1457*saec *attachment was reduced by 31% and 48%, respectively (Figure 7).

### Effect of *saeRS *deletion on PIA production and Aap expression of *S. epidermidis*

PIA in the extracellular matrix of biofilms was detected using a dot blot assay with the WGA-HRP conjugate. PIA production levels were not significantly different in the SE1457*ΔsaeRS *strain compared to the SE1457 and SE1457*saec *strains (Additional file 2: Fig. S2). When assessed by comparative proteomic analysis, expression of accumulation-associated protein (Aap), an important factor for intercellular adhesion, was up-regulated in SE1457*ΔsaeRS *compared to the wild-type strain (Additional File 3: Fig. S3). Aap in lysostaphin-treated whole bacterial lysates of SE1457*ΔsaeRS*, SE1457 and SE1457*saec *strains was detected by Western blot using an anti-Aap monoclonal antibody. The SE1457*ΔsaeRS *strain expressed more Aap (1.85-fold up-regulation) compared to the wild-type and the complementation strains (Additional file 4: Fig. S4).

### Analysis of the autolysis-related gene transcription in SE1457*ΔsaeRS*

To investigate whether the transcription of autolysis-related genes was regulated by *saeRS*, DNA microarray and RT-qPCR of total RNAs from the SE1457*ΔsaeRS *and the wild-type strains were performed. Expression of numerous autolysis-related genes including *lytS *(two-component sensor histidine kinase LytS), *lrgA *(holin-like protein), *serp0043 *(1,4-beta-N-acetylmuramidase), *glpQ *(glycerophosphoryl diester phosphodiesterase), *arlR *(DNA-binding response regulator)*, atlE *(autolysin), and *aae *(autolysin/adhesin) was found to be up-regulated in SE1457*ΔsaeRS *strain (Table 3). Transcription of the genes in the *saeRS *operon (*saeP, saeQ, saeR*, and *saeS*) in the SE1457*ΔsaeRS *strain was not detected.

**Table 3:** Genes expression regulated by *saeRS *in *S. epidermidis*

Genbank accession no.	Genes/ORF	Description	Expression ratio mutant/WT		P-value^b^	Functions	References
						
			Microarray^a ^	RT-qPCR			
Autolysis-related genes							
AAW52842	*lytS*	two-component sensor histidine kinase LytS	3.87	2.33 ± 0.35	0.0097	Negatively modulating the expression of murein hydrolases and positively regulates the expression of the *lrgAB *operon in *S. aureus*	[27,43,44]
AAW52844	*lrgA*	holin-like protein LrgA	2.28	2.75 ± 0.05	< 0.0001	Encoding a murein hydrolase exporter similar to bacteriophage holin proteins; may be required for the activity or transport of this cell wall-associated murein hydrolase in *S. aureus*	[44]
AAW53428	*serp0043*	1,4-beta-N-acetylmuramidase	4.86	2.25 ± 0.20	0.0016	Having lysozyme activity in peptidoglycan catabolic process in *S. aureus*	[14]
AAW53918	*glpQ*	glycerophosphoryl diester phosphodiesterase GlpQ, putative	2.98	1.80 ± 0.20	0.0080	Having glycerophosphodiester phosphodiesterase activity in lipid and glycerol metabolic process in *S. aureus*	[55]
AAW54343	*arlR*	DNA-binding response regulator	8.30	3.20 ± 0.45	0.0015	Regulating extracellular proteolytic activity; may be involved in the modulation of expression of genes associated with growth and cell division; positively regulating a two-component system *lytRS *in *S. aureus*	[18,25,26,56-58]
AAW53968	*atlE*	*S. epidermidis *autolysin	UD^c^	1.45 ± 0.10	0.0053	Having amidase activity to cleave the amide bond between N-acetyl muramic acid and L-alanine; mediating lysis of a subpopulation of the bacteria and extracellular DNA release in *S. epidermidis *	[7,29,46]
AJ250905	*aae*	*S. epidermidis *autolysin/adhesin	UD	2.32 ± 0.38	0.0088	Having bacteriolytic activity and binding to fibrinogen, fibronectin and vitronectin in *S. epidermidis*	[8]
Biofilm-forming related genes							
AAW53175	*icaA*	a gene of ica operon	UD	1.22 ± 0.13	0.20	Encoding N-acetyglucosaminyltransferase for synthesis of polysaccharide intercellular adhesin (PIA) which is important for biofilm formation of *S. epidermidis*	[2,31,59]
AAW53239	*aap*	accumulation-associated protein	UD	1.62 ± 0.06	0.0008	Contributing to intercellular adhesion and biofilm formation of *S. epidermidis*	[4,60,61]
sae operon							
AAW53762	*saeS*	sensor histidine kinase SaeS	0.26	UD		Encoding a histidine kinase; involving in the tight temporal control of virulence factor expression in *S. aureus*	[18,47,62]
AAW53763	*saeR*	DNA-binding response regulator SaeR	0.14	UD		The response regulator SaeR binding to a direct repeat sequence in *S. aureus*; involving in anaerobic growth and nitrate utilization in *S. epidermidis*	[11,48]
AAW53764	*saeQ*	conserved hypothetical protein	UD	UD		Encoding a membrane protein, function unknown in *S. epidermidis*	[62]
AAW53765	*saeP*	lipoprotein, putative	UD	UD		Encoding a lipoprotein, function unknown in *S. epidermidis*	[62]

a The complete raw microarray dataset has been posted on the Gene Expression Omnibus database (http://www.ncbi.nlm.nih.gov/geo/), accession number GPL13532 for the platform design and GSE29309 for the original dataset.

b P-values of RT-qPCR results were caculated using Student's t-test.

c UD: under detection level in microarray analysis or by RT-qPCR.

## Discussion

As *Staphylococci *biofilm formation is influenced by external factors such as glucose, NaCl, temperature, aerobiosis-anaerobiosis, static-dynamic conditions, and pH [36-39], it suggests that there are mechanisms that can sense environmental signals and regulate bacterial biofilm formation. In *S. epidermidis*, the *agrC*/*A *TCS has been proven to negatively regulate biofilm formation [15,16], while the *lytS/R *TCS has been shown to positively regulate bacterial autolysis [40]. In *S. aureus*, the *saeRS *TCS influences biofilm formation [17] and the expression of virulence-associated factors [18], whereas in *S. epidermidis*, a mutant with *saeR *deletion showed a slightly higher biofilm-forming ability compared to the parental strain [11].

In the present study, SE1457*ΔsaeRS*, a *saeR *and *saeS *deletion mutant from *S. epidermidis *1457, was constructed by homologous recombination. Although *saeRS *in *S. epidermidis *ATCC 35984 and *S. aureus *Newman are similar both at nucleotide sequence level (75% for *saeR *and 67% for *saeS*) and at the amino acid level (84% for SaeR and 70% for SaeS), both biofilm formation and autolysis were up-regulated in SE1457*ΔsaeRS*, suggesting that *saeRS *in *S. epidermidis *plays a different role from that in *S. aureus*. Additionally, when examined by SEM, increased quantities of extracellular polymeric substances (EPSs) were observed in the SE1457*ΔsaeRS *biofilm compared to the SE1457 and SE1457*saec *biofilms (Figure 2A).

Aap expression and PIA synthesis are important for biofilm formation. Therefore, we examined the contribution of Aap and PIA to SE1457*ΔsaeRS *biofilm formation. In *S. epidermidis*, Aap plays an important role in biofilm formation, and biofilm-positive strains that express *aap *show higher biofilm forming abilities than strains that lack the Aap protein [41]. In SE1457*ΔsaeRS*, Aap up-regulation was detected using 2-DE and confirmed by Western blot, suggesting that Aap is a factor associated with the enhanced biofilm formation capacity of SE1457*ΔsaeRS*. PIA plays a major role in intercellular adhesion in *S. epidermidis *biofilms [42]. However, no obvious differences in either PIA production or transcription of *icaA*, the gene that encodes an N-acetylglucosaminyl transferase enzyme critical for PIA synthesis, were observed between SE1457*ΔsaeRS *and SE1457 (Table 3). These results are consistent with the findings reported for a *saeR *deletion mutant by Handke *et al*. [11].

The enhanced *S. epidermidis *biofilm formation may be correlated with the increased amounts of eDNA released in the biofilm matrix [19,25,28]. Quantitative PCR revealed that eDNA release from *S. epidermidis *1457*ΔsaeRS *was up-regulated (Figure 6). Furthermore, the biomass of SE1457*ΔsaeRS *biofilms was markedly decreased compared to SE1457 and SE1457*saec *biofilms when DNase I was added prior to biofilm formation.

Extracellular DNA is known to be released following bacterial autolysis [19]. SE1457*ΔsaeRS *showed a higher level of Triton X-100-induced autolysis compared to the wild-type strain in TSB medium containing 1 M NaCl. In accordance with the enhanced autolysis of SE1457*ΔsaeRS*, extracts from SDS-treated SE1457*ΔsaeRS *cells exhibited more bacteriolytic bands compared to extracts from the wild-type strain. These results indicate that *saeRS *influenced the activity of autolysins that bind non-covalently to the *S. epidermidis *cell wall. In *S. aureus*, autolysis is a complicated process regulated by the *lytSR *TCS [43] and global regulators such as *mgrA *and *sarA *[44,45]. Autolysis is influenced by a variety of different factors such as NaCl, pH, temperature, and growth phase, suggesting the existence of a mechanism that can sense environmental conditions [36-39]. However, Zhu et al. have demonstrated that the *lytSR *TCS in *S. epidermidis *is not involved in Triton X-100-induced autolysis and does not alter the zymogram profile [40], indicating that a different mechanism for autolysis regulation exists in *S. epidermidis*. The findings in the present study suggest that the *saeRS *TCS may regulate *S. epidermidis *autolysis.

The increased autolysis rate observed in SE1457*ΔsaeRS *may also be associated with the up-regulated expression of autolysins. In *S. epidermidis*, AtlE and Aae are important autolysins [8,46]. AtlE is expressed as a 138 kDa precursor protein that is proteolytically processed to release the GL (51 kDa) and AM domains (62 kDa) [13,14,23]. Aae, a 35 kDa protein, contains three repetitive sequences in its N-terminal portion. These repeats comprise features of a putative peptidoglycan binding domain (LysM domain) found in several enzymes that are involved in cell-wall metabolism. Aae from *S. epidermidis *O-47 exhibited bacteriolytic activity in zymographic analysis using *S. carnosus *or *S. epidermidis *cells as a substrate. In the present study, *atlE *and *aae *transcription was up-regulated in SE1457*ΔsaeRS *(Table 3), which may account for the increase in bacteriolytic bands in the zymogram assay. In addition, expression of numerous autolysis-related genes in SE1457*ΔsaeRS*, such as *lytS, lrgA, arlR, serp0043 *and *glpQ*, were also up-regulated, suggesting that *S. epidermidis *autolysis mediated by *saeRS *may be influenced by other factors that remain to be defined.

Transcriptional profile analysis of the *saeRS *mutant and the wild-type strain found 135 differentially expressed genes in the present study, whereas in the Handke's study, only 65 genes in the *saeR *mutant were differentially expressed compared to the wild-type strain. The deletion of *saeRS *in *S. epidermidis *affects genes with a variety of functions, including bacterial autolysis (*lrgA*, *arlR, lytS*), biofilm formation (*ebhA*), leucine biosynthesis (*leuD*), protein hydrolysis (*clpP*), stress resistance (*asp23*), and cell viability (*yycH*). Three genes with increased expression, *pflB *(formate acetyltransferase), *pflA *(formate acetyltransferase-activating enzyme) and *lrgA *(holin protein) in SE1457*ΔsaeRS*, overlapped with the *saeR *deletion mutant. The discrepancies of the microarray data between the *saeR *mutant and the *saeRS *mutant may result from crosstalk between *saeS *and the response regulators of other TCSs. When the transcriptional profiles of the *saeRS *deletion mutant was compared to the *S. aureus *strains N315, COL, and Newman, only three differentially expressed genes, *geh *(glycerol ester hydrolase), *efb *(fibrinogen-binding protein) and *lrgA *(holin-like protein LrgA), were found to overlap [18,47]. Taken together, these results suggest a different role for *saeRS *in *S. epidermidis *from that in *S. aureus*.

Through the use of regulatory sequence analysis tools (http://rsat.ulb.ac.be/rsat), we further analyzed the upstream regions of the genes that were differentially expressed in SE1457*ΔsaeRS *compared to the wild-type strain for the GTTAAN6GTTAA SaeR-binding motif in *S. aureus *reported by Sun et al. [48]. Only Eight genes involved in metabolic process [SERP2414, SERP2360, SERP2192 (*cysH*), SERP1745 (*deoC*), SERP0721 (*pheS*), SERP0371, SERP0365 (*saeR*), and SERP0164] that contained the direct repeat sequence with no more than one mismatch were found (Table 4), suggesting that the potential role of *saeRS *in autolysis regulation in *S. epidermidis *may be different from its role in *S. aureus*.

**Table 4:** Genes containing the direct repeat sequence with no more than one mismatch

Gene ID^a^	Name	Start^b^	Sequence^c^	End^b^	Product
SERP0164		-1	GTTAAATTTAATTTAA	-16	ATP:guanido phosphotransferase family protein
SERP0365	*saeR*	-488	GTTAAATCATATTTAA	-503	DNA-binding response regulator SaeR
SERP0371		-575	GTTAATCTTCATTTAA	-590	exsD protein
SERP0721	*pheS*	-648	GATAACATGATGTTAA	-663	phenylalanyl-tRNA synthetase, alpha subunit
SERP1745	*deoC*	-1091	GTAAAAATAAAGTTAA	-1106	deoxyribose-phosphate aldolase
SERP2192	*cysH*	-172	GATAATCAAAAGTTAA	-187	phosophoadenylyl-sulfate reductase
SERP2360		-114	GTTAAACCACCGTCAA	-129	3-hydroxyacyl-CoA dehydrogenase family protein
SERP2414		-270	GTTAACAGATAGTAAA	-285	lipoprotein, putative

a These genes are identified in microarray analysis.

b The start point and end point are the distance from the translation start codon.

c Conserved repeat sequences are underlined.

## Conclusions

The deletion of *saeRS *in *S. epidermidis *resulted in the alteration of bacterial autolysis, increased eDNA release, and decreased bacterial cell viability in the planktonic/biofilm states. Further, Aap expression and the transcription of autolysin genes such as *atlE *and *aae *were up-regulated. Overall, these alterations were associated with the increased biofilm-forming ability of the *saeRS *deletion mutant. The present study suggests that in *S. epidermidis*, the *saeRS *TCS plays an important role in regulating bacterial autolysis, which is related to biofilm formation.

## Competing interests

The authors declare that they have no competing interests.

## Authors' contributions

QL performed the molecular genetic studies, participated in the sequence alignment, and drafted the manuscript. TZ helped to construct the *saeRS *deletion mutant. JH performed the autolysis and zymogram analysis. HB participated in the 2-DE study. JY performed the RT-qPCR analysis. FY participated in the CLSM analysis. JL participated in the RNA extractions. YW participated in the design of the study, performed the statistical analysis and edited the manuscript. AF, PF, and JS performed and analyzed microarray experiments. DQ participated in the study design and coordination and helped to draft and edit the manuscript. All authors read and approved the final manuscript.

## Supplementary Material

Additional file 1 **Fig. S1. Growth curves of SE1457*ΔsaeRS *and the parental strain in aerobic (A) or anaerobic (B) growth conditions**. Overnight cultures were diluted 1:200 and incubated at 37°C with shaking at 220 rpm. The OD600 of the cultures was measured at 60 min intervals for 12 h. For anaerobic growth conditions, bacteria were cultured in the Eppendorf tubes that were filled up with the TSB medium and sealed with wax. WT, SE1457; SAE, SE1457*ΔsaeRS*. Click here for file

Additional file 2 **Fig. S2. PIA detection in *S. epidermidis *biofilms**. *S. epidermidis *strains were grown in 6-well plates under static conditions at 37°C for 24 h. Next, the cells were removed by scraping and collected by centrifugation before being resuspended in 0.5 M EDTA (pH 8.0). After proteinase K treatment (20 mg/mL) for 3 h at 37°C, serial dilutions of the PIA extracts were spotted onto PVDF membranes. Spots corresponding to PIA were quantified using the Quantity-one software. WT, SE1457; SAE, SE1457*ΔsaeRS*; SAEC, SE1457*saec*; 35984, *S. epidermidis *ATCC35984. Click here for file

Additional file 3 **Fig. S3. SE1457*ΔsaeRS *and wild-type strain 2-DE profiles**. SE1457*ΔsaeRS *and SE1457 were grown in TSB medium at 37°C until the post-exponential growth phase; the bacteria were then separated by centrifugation. Bacteria cell pellets were dissolved in lysis buffer and sonicated on ice. The 2-DE gels were performed using 24 cm immobilized dry strips (IPG, nonlinear, pH 4-7, GE Healthcare) and analyzed by ImageMaster 2D platinum 6.0 software (Amersham Biosciences). Protein spots were identified using a 4700 MALDI-TOF/TOF Proteomics Analyzer (Applied Biosystems, California, USA). Click here for file

Additional file 4 **Fig. S4. Detection of Aap expression**. Aap in lysostaphin-treated bacterial cells of SE1457*ΔsaeRS*, SE1457, and SE1457*saec *was detected by Western blot using an anti-Aap monoclonal antibody (made in our laboratory). Proteins were separated on 7% SDS-PAGE gels and then transferred to polyvinylidene fluoride (PVDF) membranes by electroblotting. Bands corresponding to Aap were quantified using the Quantity-one software. WT, SE1457; SAE, SE1457*ΔsaeRS*; SAEC, SE1457*sae.* Click here for file
