# Supplementary figures and images for: Role of the SaeRS two-component regulatory system in Staphylococcus epidermidis autolysis and biofilm formation
Source: BMC Microbiol. 2011 Jun 24;11:146. doi: 10.1186/1471-2180-11-146 (PMC3224141; doi:10.1186/1471-2180-11-146)

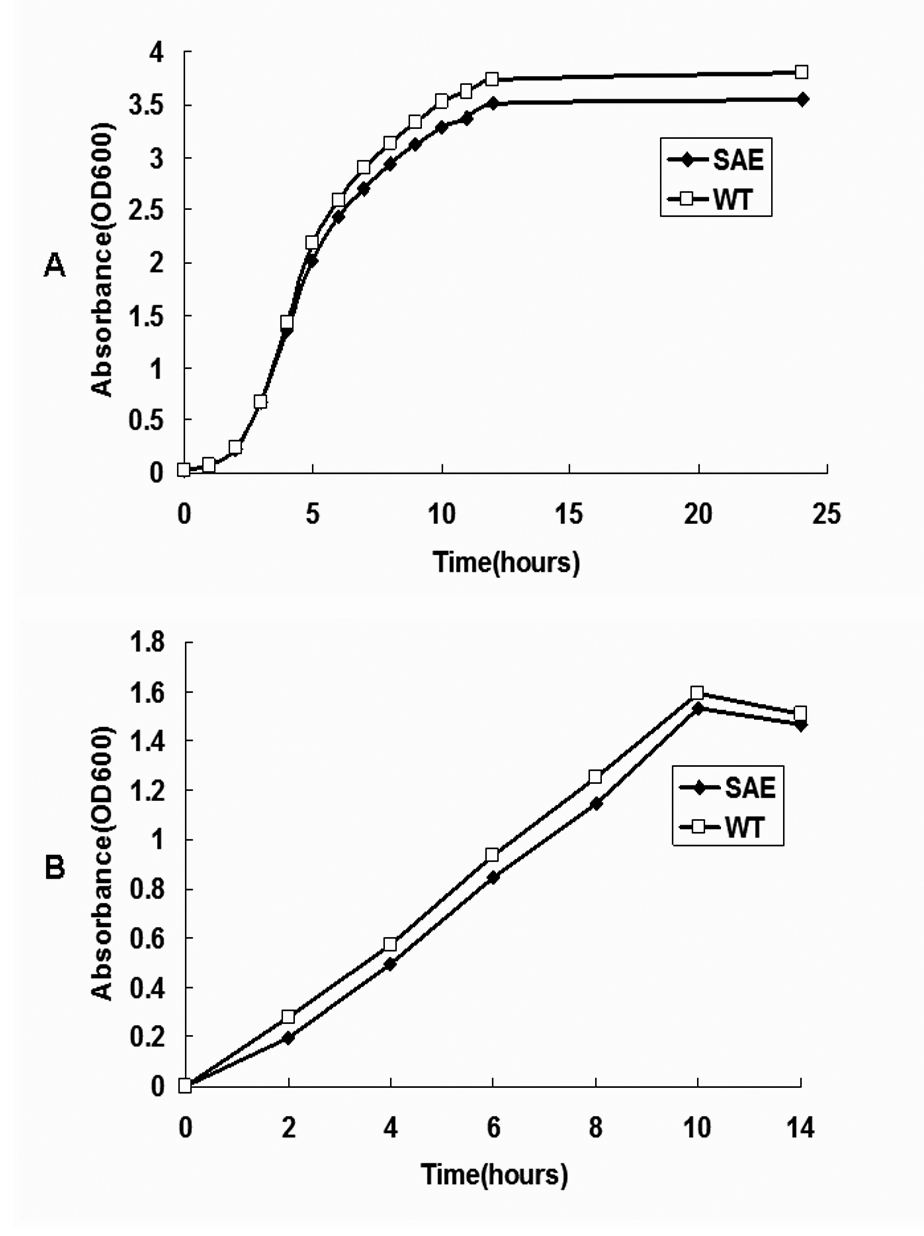

Supplement: Additional file 1 — Fig. S1. Growth curves of SE1457ΔsaeRS and the parental strain in aerobic (A) or anaerobic (B) growth conditions. Overnight cultures were diluted 1:200 and incubated at 37°C with shaking at 220 rpm. The OD600 of the cultures was measured at 60 min intervals for 12 h. For anaerobic growth conditions, bacteria were cultured in the Eppendorf tubes that were filled up with the TSB medium and sealed with wax. WT, SE1457; SAE, SE1457ΔsaeRS. [file 1471-2180-11-146-S1.TIFF]

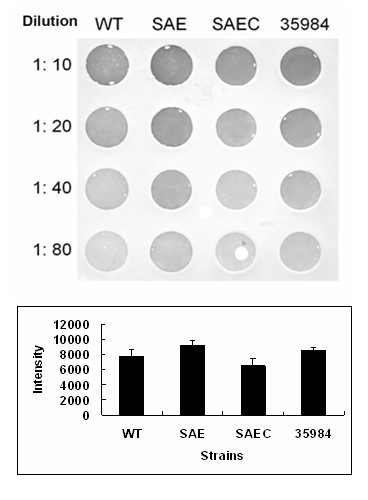

Supplement: Additional file 2 — Fig. S2. PIA detection in S. epidermidis biofilms. S. epidermidis strains were grown in 6-well plates under static conditions at 37°C for 24 h. Next, the cells were removed by scraping and collected by centrifugation before being resuspended in 0.5 M EDTA (pH 8.0). After proteinase K treatment (20 mg/mL) for 3 h at 37°C, serial dilutions of the PIA extracts were spotted onto PVDF membranes. Spots corresponding to PIA were quantified using the Quantity-one software. WT, SE1457; SAE, SE1457ΔsaeRS; SAEC, SE1457saec; 35984, S. epidermidis ATCC35984. [file 1471-2180-11-146-S2.TIFF]

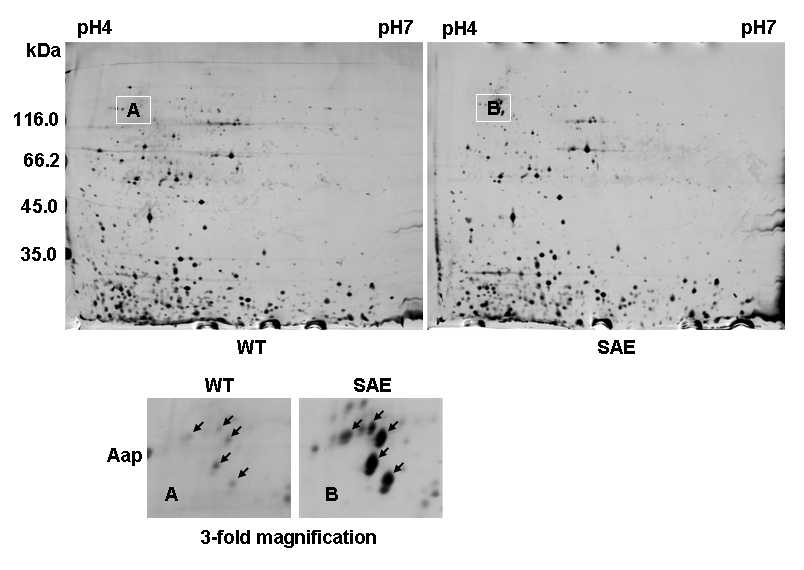

Supplement: Additional file 3 — Fig. S3. SE1457ΔsaeRS and wild-type strain 2-DE profiles. SE1457ΔsaeRS and SE1457 were grown in TSB medium at 37°C until the post-exponential growth phase; the bacteria were then separated by centrifugation. Bacteria cell pellets were dissolved in lysis buffer and sonicated on ice. The 2-DE gels were performed using 24 cm immobilized dry strips (IPG, nonlinear, pH 4-7, GE Healthcare) and analyzed by ImageMaster 2D platinum 6.0 software (Amersham Biosciences). Protein spots were identified using a 4700 MALDI-TOF/TOF Proteomics Analyzer (Applied Biosystems, California, USA). [file 1471-2180-11-146-S3.TIFF]

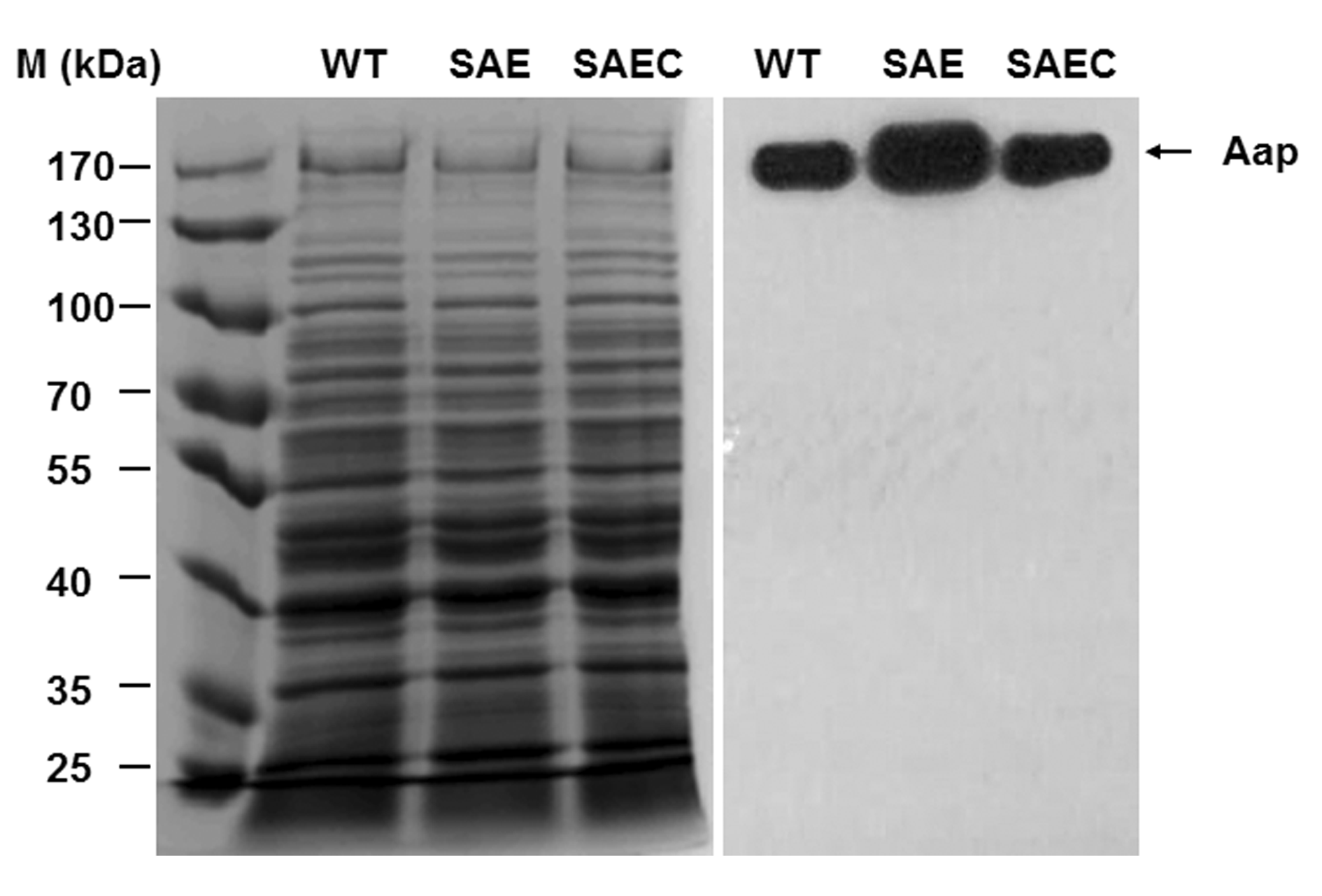

Supplement: Additional file 4 — Fig. S4. Detection of Aap expression. Aap in lysostaphin-treated bacterial cells of SE1457ΔsaeRS, SE1457, and SE1457saec was detected by Western blot using an anti-Aap monoclonal antibody (made in our laboratory). Proteins were separated on 7% SDS-PAGE gels and then transferred to polyvinylidene fluoride (PVDF) membranes by electroblotting. Bands corresponding to Aap were quantified using the Quantity-one software. WT, SE1457; SAE, SE1457ΔsaeRS; SAEC, SE1457sae. [file 1471-2180-11-146-S4.TIFF]
